# Resource loss, coping strategies and post-traumatic stress disorder symptoms in survivors of the 2020 Croatia earthquake

**DOI:** 10.1186/s40359-023-01176-5

**Published:** 2023-04-25

**Authors:** Ajana Löw, Martina Lotar Rihtarić, Ivana Vrselja

**Affiliations:** 1 Faculty of Education and Rehabilitation Sciences, University of Zagreb, University Campus Borongaj, Borongajska cesta 83f, 10000 Zagreb, Croatia; 2 Department of Psychology, Catholic University of Croatia, Zagreb, Croatia

**Keywords:** Earthquake, PTSD, Resource loss, Conservation of resources theory, Coping

## Abstract

**Background:**

Conservation of resources theory (COR) establishes a link between resource loss and the stress response. The aim of this study was to assess the contribution of resource loss in the form of home damage and the choice of active or passive coping strategies to PTSD symptoms in survivors of the 2020 Petrinja (Croatia) earthquake.

**Methods:**

A total of 374 adults (29.9% men) aged 18–64 years living in the counties surrounding the epicenter of the Petrinja (Croatia) earthquake participated in an online cross-sectional survey. The questionnaire included the PTSD Checklist for DSM-5 (PCL-5), the Coping Inventory, and the binary item assessing whether or not the participants' home was damaged.

**Results:**

Hierarchical regression analysis showed that home damage was a significant predictor of PTSD symptoms. Participants whose homes were damaged by the earthquake were significantly more likely to use passive coping strategies, namely avoidance and emotional venting, and one active coping strategy, action, than those whose homes were spared. Finally, more frequent use of passive coping was associated with a higher risk of PTSD symptoms.

**Conclusions:**

The study corroborates the COR theory link between resource loss and the stress response, as well as the general consensus that passive coping is a less adaptive strategy than active coping. In addition to passive coping, individuals who lacked resources may have been inclined to take some active steps because they either needed to repair or relocate their homes and because most buildings were only moderately to minimally damaged in the Petrinja earthquake.

## Introduction

On December 29, 2020, at 12 h and 19 min, the Croatian population was shaken by a strong earthquake with the epicenter near Petrinja (Sisačko-Moslavačka County) with a magnitude of 6.2 on the Richter scale and almost VIII-IX on the European Macroseismic Scale. Strong shaking was also felt in neighboring counties, including the capital city of Zagreb, and moderate shaking was felt throughout the country. There were seven fatalities, six people were seriously injured, about twenty people were slightly injured, and approximately 15% of buildings in towns near the epicenter were severely damaged or demolished [1, 2]. In the three days following the main earthquake, there were more than 2000 aftershocks, and nearly 1000 people were evacuated to shelters. Together with the Zagreb earthquake in March 2020 (5.5 on the Richter scale), seismic activity in Croatia has greatly increased after a long period of inactivity that lasted almost a century.

Earthquake disasters usually include a main earthquake and many aftershocks, which can occur days, weeks, months, or years after the main event, evoking memories of the original event [3]. Furthermore, because some earthquakes begin with a foreshock, one can never be sure if there will be an even stronger quake. Since natural disasters such as earthquakes are usually sudden and unpredictable, survivors often experience negative psychological consequences that spill over into other areas of life [4, 5]. In a qualitative study following the 2010 Christchurch (New Zealand) earthquake, participants reported multiple responses to the earthquake and aftershocks, ranging from immediate to persistent fear, anxiety and constant hypervigilance, inability to “move forward”, concerns regarding financial hardship and even sadness [6].

A meta-analysis of 52 studies investigating psychological effects on survivors after different natural disasters found that although disasters have a negative impact on mental health and can lead to various types of psychopathology, these effects weaken over time [7]. However, the unique combination of persistent aftershocks and uncertainty about the potential occurrence of another, possibly more powerful earthquake distinguishes earthquakes from other types of disasters, such as floods, wildfires, or severe storms. Thus, stress after an earthquake may not decrease over time but instead transition from acute to chronic, with significant long-term effects on mental health [8]. Indeed, there is clear evidence that earthquake stress is more emotionally devastating than stress arising from one-time disasters [9], and that the anxiety and fear associated with earthquake exposure can last for years [10, 11]. The long-term psychological effects of earthquakes can be exacerbated by the relatively high numbers of injuries and deaths involved, the potentially extensive destruction of homes, and lack of organized support from social services in the aftermath [9]. These considerations highlight the need to understand how survivors of earthquakes cope with the mental strain.

### Theoretical framework: conservation of resources theory

An approach that may help in this regard is conservation of resources theory (COR) [12–14], a comprehensive stress and coping theory which posits that individuals are naturally motivated to acquire and maintain their resources, i.e. valued entities that include objects, attributes, or states. Although not in contrast to traditional models of stress [15], COR encompasses both individual and environmental factors, highlighting the fit of resources and environmental demands as a key factor in the stress response. The COR theory consists of several principles and corollaries that can be summarized as follows: (1) loss of resources has stronger psychological consequences than gain of resources, (2) individuals must maintain or invest their resources in order to protect against or recover from their loss, (3) individuals who lack resources are more vulnerable to further loss of resources, and (4) individuals who lack resources are likely to use defensive coping strategies to maintain their resources.

The principles of COR theory have been empirically tested in the context of natural disasters. For example, among survivors of Hurricane Hugo in the United States, loss of resources predicted onset of clinical symptoms of post-traumatic stress disorder (PTSD), and it predicted psychological distress better than individual characteristics or coping strategies [16]. Similar findings were reported nearly two decades later among survivors of Hurricane Katrina in the United States [17]. Indeed, loss of resources has long-term psychological consequences that persist for several years after the disaster [18]. Such loss has been linked to increase in PTSD and depression symptoms and decrease in life satisfaction among flooding survivors [19]. The role of resource loss has also been documented in immune system changes among hurricane survivors [20] and in onset of various physical symptoms among flood survivors [21].

In analyzing the potential influence of resource loss, care should be taken to differentiate between resource loss and pre-existing resource scarcity. Resource loss refers to the loss of valued entities, such as the loss of one's home, due to the major stress or a traumatic event, such as a natural disaster [22], whereas resource scarcity refers to a person's lack of or limited access to various forms of capital, such as income, education, employment, or other financial, social, or cultural resources [23]. Although chronic resource scarcity makes people more vulnerable to resource loss, a study of women of low socioeconomic status found that resource loss, but not resource scarcity, was significantly associated with depressed mood [24]. Those authors concluded that resource loss might be more detrimental to mental health than a pre-existing resource deficit.

COR theory stipulates that individuals who lack resources are likely to use defensive coping strategies, which are "passive" in that they do not require effort or resources. The use of passive coping may be a natural response of individuals experiencing deprivation, as neurobiological studies indicate that passive responses, such as immobility or reducing reactivity to the environment, can help conserve energy and preserve existing scarce resources [25]. However, researchers generally agree that active coping with stressors, in which efforts are made to solve a problem, is more adaptive in the long run than passive coping [26]. In one of the few empirical studies of passive coping in the context of natural disasters, strategies such as avoidance or self-blame correlated with poorer mental health among survivors of the 2008 Wenchuan (China) earthquake [27]. Likewise, survivors of the 2009 L'Aquila (Italy) earthquake who used more passive coping strategies (denial, venting, reduced behavioral effort, self-blame) were more likely to suffer PTSD than those who used active coping strategies [28].

### Study hypotheses

Although associated with a variety of losses, one of the most devastating consequences of earthquake disasters is property damage and the resulting loss of housing. Based upon the link in COR theory between the resources available to an individual and that individual’s ability to cope with stress [12–14], we hypothesized that loss of resources in the form of damage to one’s home would be a significant predictor of PTSD symptoms among survivors of the Petrinja earthquake (Hypothesis 1). COR also proposes that individuals who lack resources are likely to use passive coping strategies to maintain their resources [12–14]. Although the entire sample had reduced resources in the pre-earthquake period, immediate losses were greater for participants who suffered property damage. Because the element of resource loss has additional implications for mental health beyond general lack of resources [24], we hypothesized that Petrinja survivors whose houses were damaged by the earthquake would be more likely to use passive coping strategies than participants whose houses were not damaged (Hypothesis 2). Further, researchers generally agree that active coping is more adaptive than passive coping and that passive coping can have negative long-term consequences for an individual's development [26]. Therefore, we predicted that passive, but not active, coping strategies would significantly predict PTSD symptoms among Petrinja survivors (Hypothesis 3).

## Methods

### Participants

Adults living in the counties of Croatia surrounding the epicenter of the Petrinja earthquake (Sisačko-Moslavačka County, City of Zagreb, Zagreb County, Karlovac County, and Krapinsko-Zagorska County) were invited to participate in an online, cross-sectional survey. A total of 374 participants (29.9% men) between 18 and 64 years old (*M* = 35.03, *SD* = 11.449) took part.

### Measures

#### PTSD symptoms

The severity of PTSD symptoms was measured using the PTSD Checklist for DSM-5 (PCL-5; Weathers et al. [29]). This scale consists of 20 items measuring the 20 DSM-5 symptoms of PTSD. Participants were asked to rate, in relation to the Petrinja earthquake in late 2020, the extent to which each of the listed symptoms had caused them problems in the past month, based on a 5-point scale (0 = "not at all", 4 = "extremely"). The total symptom severity score was the sum of the scores for the individual items, so it ranged from 0 to 80. The scale is effective for diagnosing PTSD at scores of 33 and higher [30–32]. The PCL-5 has shown good convergent and divergent validity and high internal consistency (α = 0.91–0.95) [32, 33], but also test–retest reliability (r = 0.82) [33]. Internal consistency of the scale in the present study was α = 0.96.

#### Coping strategies

Coping strategies were analyzed using the Coping Inventory [34]. The instrument consists of 36 items that measure eight dimensions of reactions to the stressful event: action (7 items), rational thinking (5 items), positive thinking (4 items), emotional venting (6 items), instrumental support (3 items), emotional support (4 items), avoidance (4 items) and denial (3 items). Confirmatory factor analysis showed that these eight dimensions can be grouped into three higher-order factors: active coping (action, rational thinking, positive thinking), expressive support seeking (emotional venting, instrumental support, emotional support), and avoidance (avoidance, denial). The active coping factor includes reactions to a stressful event in which a person relies on his or her own strengths and resources. Expressive support seeking and avoidance include reactions that can be considered passive because a person does not rely on his or her own strengths to solve problems but relies on the support of others for stress resolution [35]. The participants were asked to estimate the frequency of the listed reactions to the stressful event after the earthquake on a 7-point scale (1 = "not at all", 7 = "a lot"). The total score was calculated as the mean of the items for each coping dimension, with a higher score indicating a greater frequency of responses within a given dimension. In the present study, α was ranging from 0.86 for rational thinking to 0.95 for action.

#### Home damage

The loss of resources in this study was defined as damage to participants' homes. Respondents were asked “Was your residential building damaged by the earthquake?”, and possible responses were “yes” or “no”. Thus, we did not differentiate among participants based on extent of home damage.

### Procedure

The survey was conducted from June 7th to September 1st, 2021. Adults who lived in counties surrounding the epicenter at the time of the earthquake were included in the research. Ethical approval was obtained from the Ethics Committee of the Catholic University of Croatia. Prior to filling out the survey, participants were informed about the purpose of the study and about how their responses would be kept confidential and anonymous. If they provided consent, they were allowed to access the entire survey, which contained items about PTSD symptoms, coping strategies, and loss of resources.

### Statistical analysis

Data were analyzed using SPSS 23 software, and statistical tests were chosen based on whether the data were normally distributed or skewed (using absolute values of skewness and kurtosis or their z-values) and considering sample or subgroups size, as suggested by Kim [36]. Differences in coping strategies between participants whose homes were damaged and participants whose homes were not damaged were assessed using the t-test or Mann–Whitney test, as appropriate.

Pearson's coefficient was used to assess potential correlations between continuous variables, while the point-biserial coefficient was used to assess correlations between continuous variables and the binary variable (home damage). Hierarchical multiple regression was performed as follows in order to identify factors associated with PTSD. In the first step, age and gender were controlled. Home damage was inserted in the second step, followed by active coping strategies in the third step, emotional support seeking coping strategies in the fourth step, and avoidant coping strategies in the fifth step. The significance level was set at *p* < 0.05.

## Results

Results on PTSD Checklist for DSM-5 have shown that 16.3% achieves score of 33 or higher. This result suggests that those participants have a higher probability of being diagnosed with PTSD [30–32]. Most coping dimensions were used to similar extents in the sample, except for denial, which was used least often (Table 1). Active coping dimensions (action, rational thinking, and positive thinking) were used slightly more often than other dimensions. Participants whose homes were damaged by the Petrinja earthquake used action, emotional venting, and avoidance significantly more often than those whose homes were spared (Table 2).

**Table 1:** Descriptive statistics for PTSD symptoms and coping dimensions (N = 374)

Variable	*M*	*SD*	Min	Max	Skewness	Kurtosis
PTSD symptoms	15.59	16.439	0	74	1.241	1.156
Action	4.80	1.496	1	7	− 0.502	− 0.313
Rational thinking	4.79	1.363	1	7	− 0.497	− 0.098
Positive thinking	4.47	1.630	1	7	− 0.365	− 0.643
Emotional venting	4.25	1.439	1	7	− 0.227	− 0.438
Instrumental support	4.04	1.744	1	7	− 0.155	− 0.864
Emotional support	4.05	1.657	1	7	− 0.066	− 0.823
Avoidance	4.36	1.612	1	7	− 0.375	− 0.471
Denial	1.77	1.356	1	7	1.916	3.111

*M* mean, *SD* standard deviation, *Min* the lowest observation, *Max* the highest observation

**Table 2:** Differences in coping dimensions between participants whose homes were damaged or not by the earthquake

Variable	Home spared (*N* = 235)			Home damaged (*N* = 139)			
	Central tendency (dispersion)	Distribution		Central tendency (dispersion)	Distribution		Test
	*M *(*SD*)	*Z*_skewness_	*Z*_kurtosis_	*M (SD)*	*Z*_skewness_	*Z*_kurtosis_	*t*
Action	4.64 (1.549)	− 2.453	− 1.579	5.07 (1.366)	− 3.204	0.409	− 2.724**
Rational thinking	4.73 (1.416)	− 2.484	− 0.845	4.90 (1.265)	− 3.306	0.809	− 1.170
Positive thinking	4.43 (1.613)	− 2.069	− 1.842	4.53 (1.660)	− 0.2.107	− 1.723	− 0.612
Emotional venting	4.12 (1.450)	− 0.918	− 1.402	4.48 (1.371)	− 1.699	0.819	− 2.366*
Instrumental support	3.91 (1.769)	− 0.213	− 2.934	4.27 (1.681)	− 1.738	− 1.485	− 1.956
Emotional support	3.95 (1.693)	− 0.277	− 2.734	4.22 (1.681)	− 0.325	− 1.882	− 1.545
Avoidance	4.13 (1.623)	− 1.572	− 1.725	4.75 (1.522)	− 2.903	− 0.211	− 3.645**

	*C *(*Q*)	*Z*_skewness_	*Z*_kurtosis_	*C *(*Q*)	*Z*_skewness_	*Z*_kurtosis_	*U*
Denial	1.00 (1.00)	11.314	8.725	1.00 (1.00)	10.345	9.539	15,907.50

*M* mean, *SD* standard deviation, *C *median, *Q *interquartile range, *t *t-ratio, *U *Mann–Whitney U

*p < 0.05; **p < 0.01

Denial in the group of those whose home was spared and those whose home was damaged deviated significantly from the normal distribution, and for this variable Table 2 shows the median and interquartile range. Therefore, the difference in denial between the two groups was tested using the Mann–Whitney test.

PTSD symptoms showed significant positive associations with home damage and with the following dimensions of passive coping: emotional venting, instrumental support, emotional support, avoidance and denial (Table 3). PTSD symptoms did not show significant associations with active coping dimensions.

**Table 3:** Pearson’s or point-biserial correlations of PTSD symptoms with home damage^a^ or coping dimensions (N = 374)

Variable	2	3	4	5	6	7	8	9	10
1. PTSD symptoms	0.19**	0.08	− 0.02	0.05	0.20**	0.32**	0.33**	0.39**	0.37**
2. Home damage	–	0.14**	0.06	0.03	0.12*	0.10	0.08	0.19*	− 0.02
3. Action		–	0.69**	0.41**	0.35**	0.33**	0.21**	0.26**	− 0.04
4. Rational thinking			–	0.47**	0.24**	0.18**	0.04	0.22**	− 0.04
5. Positive thinking				–	0.26**	0.25**	0.16**	0.35**	0.12*
6. Emotional venting					−	0.59**	0.68**	0.38**	− 0.00
7. Instrumental support						–	0.76**	0.41**	0.17**
8. Emotional support							–	0.00	0.01
9. Avoidance								–	0.21**
10. Denial									–

*p < 0.05; **p < 0.01

^a^Treated as a binary variable: 0—undamaged, 1—damaged

The predictors included in the hierarchical regression model (Table 4) explained a total of 28.5% of the observed variance in PTSD symptoms, with avoidant coping strategies (avoidance and denial) explaining the largest proportion of variance. Variables from four of five steps contributed significantly to the explained variance in PTSD symptoms; only the step that included active coping dimensions was not significant. When avoidance and denial were introduced in the final step of regression, instrumental support and emotional support were no longer significant predictors, while positive thinking became a significant negative predictor of PTSD symptoms.

**Table 4:** Hierarchical multiple regression to identify factors associated with PTSD symptoms (N = 374)

Factors	Step 1	Step 2	Step 3	Step 4	Step 5
	*β*	*β*	*β*	*β*	*β*
Age	− 0.047	− 0.055	− 0.053	− 0.017	0.029
Gender	0.217**	0.183**	0.170**	0.079	0.082
Home damage^a^		0.153**	0.148**	. 152**	0.123**
Action			0.103	0.021	0.048
Rational thinking			− 0.124	− 0.067	− 0.066
Positive thinking			0.046	0.006	− 0.109*
Emotional venting				− 0.092	− 0.034
Instrumental support				0.209*	0.100
Emotional support				0.178*	0.103
Avoidance					0.236**
Denial					0.314**
Model summary					
*R*	0.227	0.271	0.287	0.399	0.553
Adj *R*^2^	0.046	0.066	0.067	0.139	0.285
*ΔR*^2^	0.051**	0.022**	0.009	0.077**	0.146**

^a^Treated as a binary variable: 0—undamaged, 1—damaged

*p < 0.05; **p < 0.01

## Discussion

Earthquakes are more likely than other natural disasters to induce extremely strong, long-lasting feelings of anxiety and fear in survivors [11]. Croatia did not experience substantial seismic activity during the last century [1], so the earthquake near Petrinja in December 2020 caught the country relatively unprepared. The aim of the present study was to assess the contribution of home damage and choice of active or passive coping strategies to PTSD symptoms among earthquake survivors.

We hypothesized based on COR theory [12–14] that resource loss in the form of home damage would be a significant predictor of PTSD symptoms, and our data support that. Our work may provide an important extension of the COR tenet to include a situation in which the same population faces two disasters simultaneously: the Petrinja earthquake occurred when Croatia was focused on the second wave of the COVID-19 pandemic, which involved physical isolation and other measures. Such a context may have amplified stress responses to the earthquake and made coping more difficult, as suggested from analysis of survivors of the 2020 Izmir (Turkey) earthquake during the COVID-19 pandemic [37].

The coping measure used in our study includes several passive coping dimensions (emotional venting, instrumental support, emotional support, avoidance, and denial) in which a person does not rely on his or her own strengths to solve problems [34]. Our results support the COR tenet that individuals who lack resources (in our case, participants whose homes suffered damage) are more likely to use passive coping, mainly avoidance and emotional venting, than active coping. This may reflect their desire to conserve their remaining resources [25]. Our results also corroborate the finding that resource loss has additional consequences for mental health beyond general lack of resources [24]. On the other hand, we found that individuals who lack resources may also be more likely to use one dimension of active coping, namely action. At least two explanations for this result are possible. One is that after an earthquake, people need either to repair their house quickly or to move to alternative housing [28]. Therefore, survivors whose homes have been damaged may be compelled to take at least some active steps to deal with the disaster aftermath, even if they are deprived of resources. Another explanation is that of the 23 000 buildings surveyed after the Petrinja earthquake, only 13% were severely damaged, 22% were moderately damaged, and 65% were minimally damaged [1]. This implies a smaller “barrier” to undertaking actions to repair one’s home and preserve existing resources.

Our data confirmed our third hypothesis: more frequent use of passive coping was associated with greater risk of PTSD symptoms. These findings are consistent with the general consensus in the literature regarding the adaptive capacity of different coping strategies. Although passive coping strategies can be useful in certain circumstances, active coping appears to be more adaptive in the long run [26]. Among the dimensions of passive coping, avoidance and denial explained almost twice as much of the observed variance in PTSD symptoms as instrumental and emotional support did. Thus, avoiding or denying stress due to the Petrinja earthquake was associated with higher risk of PTSD symptoms than engaging in expressive support-seeking.

Instrumental support and emotional support were no longer significant predictors of PTSD symptoms after we introduced avoidance into the model. This suggests that avoidance behavior may mediate the association between expressive support-seeking and PTSD symptoms. In other words, individuals may avoid dealing directly with the problem by seeking support, which may increase risk of PTSD symptoms. This explanation is consistent with Lazarus and Folkman's [15] classification of support seeking as an emotion-oriented coping strategy rather than problem-oriented coping strategy like planning or seeking alternatives. Although seeking support is generally considered an adaptive coping strategy, this strategy often involves only short-term resolution of emotional tensions, so the person does not directly address the problem. This is captured in some statements on the Coping Inventory that we used, such as "I have sought comfort from others" or "I have relied on others to cheer me up". It should also be noted that positive thinking became a significant negative predictor of PTSD symptoms in our sample after the introduction of the avoidance coping strategy in the last step of hierarchical multiple regression. This may merely be an artifact of the regression method [38] and should be verified in future work.

There are several limitations of this study. First, the cross-sectional design does not allow us to draw conclusions about causal relationships among PTSD, damage to homes, or coping strategies. Second, our data collection occurred once at five to eight months after the earthquake. Since PTSD symptoms have been shown to have different time trajectories for different groups of people [39], it is possible that somewhat different results will be obtained at a later time point. Third, our population comprised nearly 70% women, and most participants were young adults in their 30 s. Thus, future studies should examine whether our results generalize to men, who are less likely than women to use certain coping strategies such as expressive support seeking [40], and to older people, who may be less likely than young people to use active coping strategies [41]. Moreover, participants needed access to the internet to complete our questionnaire, so we excluded survivors who did not have Internet access or did not wish to fill out the survey online, perhaps biasing our sample towards those who were younger or who suffered less damage to their homes. Fourth, we defined resource loss simply as a binary variable related to residential building damage; we did not examine the extent of the damage or other possible material or physical impacts on participants or their family members. Future studies should verify and extend our analyses longitudinally during long-term follow-up.

## Data Availability

The datasets used and/or analysed during the current study available from the corresponding author on reasonable request.
